# EPB41L4A-AS1 is required to maintain basal autophagy to modulates Aβ clearance

**DOI:** 10.1038/s41514-024-00152-6

**Published:** 2024-05-04

**Authors:** Ziqiang Wang, Ruomei Wang, Lixin Niu, Xiaoyan Zhou, Jinxiang Han, Kun Li

**Affiliations:** 1 Department of Nuclear Medicine, The First Affiliated Hospital of Shandong First Medical University & Shandong Provincial Qianfoshan Hospital, Jinan, 250014 China; 2 Biomedical Sciences College & Shandong Medicinal Biotechnology Centre, Shandong First Medical University & Shandong Academy of Medical Sciences, Jinan, 250062 China

**Keywords:** Epigenetics, Transcription

## Abstract

Alzheimer’s disease (AD) is the most common neurodegenerative disorder characterized by the deposition of β-amyloid (Aβ) plaques. Aβ is generated from the cleavage of the amyloid precursor protein by β and γ-secretases and cleared by neuroglial cells mediated autophagy. The imbalance of the intracellular Aβ generation and clearance is the causative factor for AD pathogenesis. However, the exact underlying molecular mechanisms remain unclear. Our previous study reported that EPB41L4A-AS1 is an aging-related long non-coding RNA (lncRNA) that is repressed in patients with AD. In this study, we found that downregulated EPB41L4A-AS1 in AD inhibited neuroglial cells mediated-Aβ clearance by decreasing the expression levels of multiple autophagy-related genes. We found that EPB41L4A-AS1 regulates the expression of general control of amino acid synthesis 5-like 2, an important histone acetyltransferase, thus affecting histone acetylation, crotonylation, and lactylation near the transcription start site of autophagy-related genes, ultimately influencing their transcription. Collectively, this study reveals EPB41L4A-AS1 as an AD-related lncRNA via mediating Aβ clearance and provides insights into the epigenetic regulatory mechanism of EPB41L4A-AS1 in gene expression and AD pathogenesis.

## Introduction

Alzheimer’s disease (AD) is the most common neurodegenerative disorder, characterized by progressive memory and cognitive dysfunction^1^. AD has a high incidence in the elderly population. It is expected that by 2050, a new patient will appear every 3 s worldwide. By then, more than 130 million people will suffer from the disease^2^. AD is a highly fatal disease, fourth only to heart disease, cancer, and stroke, and has become a serious threat to global public health^3^. The histopathological hallmark of this disease is the deposition of β-amyloid (Aβ) plaques and the imbalanced Aβ generation and clearance are thought to be the causative factors for AD progression^4,5^. However, the exact underlying molecular mechanisms explaining the imbalance between Aβ generation and cleavage remain unclear. Aβ is generated from the cleavage of the amyloid precursor protein by β and γ-secretases^6^ and cleared by neuroglial cells mediated autophagy^7^.

EPB41L4A-AS1 is a newly identified long non-coding RNA (lncRNA). Since its discovery in 2019, EPB41L4A-AS1 has been implicated in several pathological processes^8–15^. EPB41L4A-AS1 is abnormally expressed in a variety of cancers: it is downregulated in non-small cell lung cancer^8^ and bone marrow-derived mesenchymal stem cells^9^, and is upregulated in colorectal cancer^10^, osteosarcoma^11^, and neuroblastoma^12^. Studies have shown that EPB41L4A-AS1 expression is regulated by P53, which affects glycolysis, glutamine metabolism, and the Warburg effect^13^. In addition, EPB41L4A-AS1 expression is abnormally increased in type 2 diabetes mellitus (T2DM), which inhibits glucose uptake and mitochondrial respiration by epigenetically regulating the transcription of glucose transporter 4 and thioredoxin-interacting proteins^14^. In a previous study, we found that EPB41L4A-AS1 is an aging-related lncRNA that is significantly downregulated in patients with AD^15^.

In the current study, to understand the potential roles of EPB41L4A-AS1 in AD progression, we analyzed its expression levels in different brain regions and at different Braak stages of patients with AD and normal controls using AD datasets (GSE5281 and GSE48350). We found that EPB41L4A-AS1 downregulation in patients with AD inhibited the clearance of Aβ by regulating the transcriptional activities of multiple autophagy-related genes. Specifically, we characterized the functional significance of EPB41L4A-AS1 in modulating histone modification and found that silencing EPB41L4A-AS1 expression altered histone acetylation, crotonylation, and lactylation located near the transcription start sites (TSSs) of these autophagy-related gene promoters by downregulating the expression of the general control of amino acid synthesis 5-like 2 (GCN5L2). Collectively, our results reveal that EPB41L4A-AS1 is involved in the clearance of Aβ through epigenetic regulation of autophagy-related genes.

## Results

### EPB41L4A-AS1 is an AD-related lncRNA associated with multiple autophagy-related genes

**Fig. 1:**
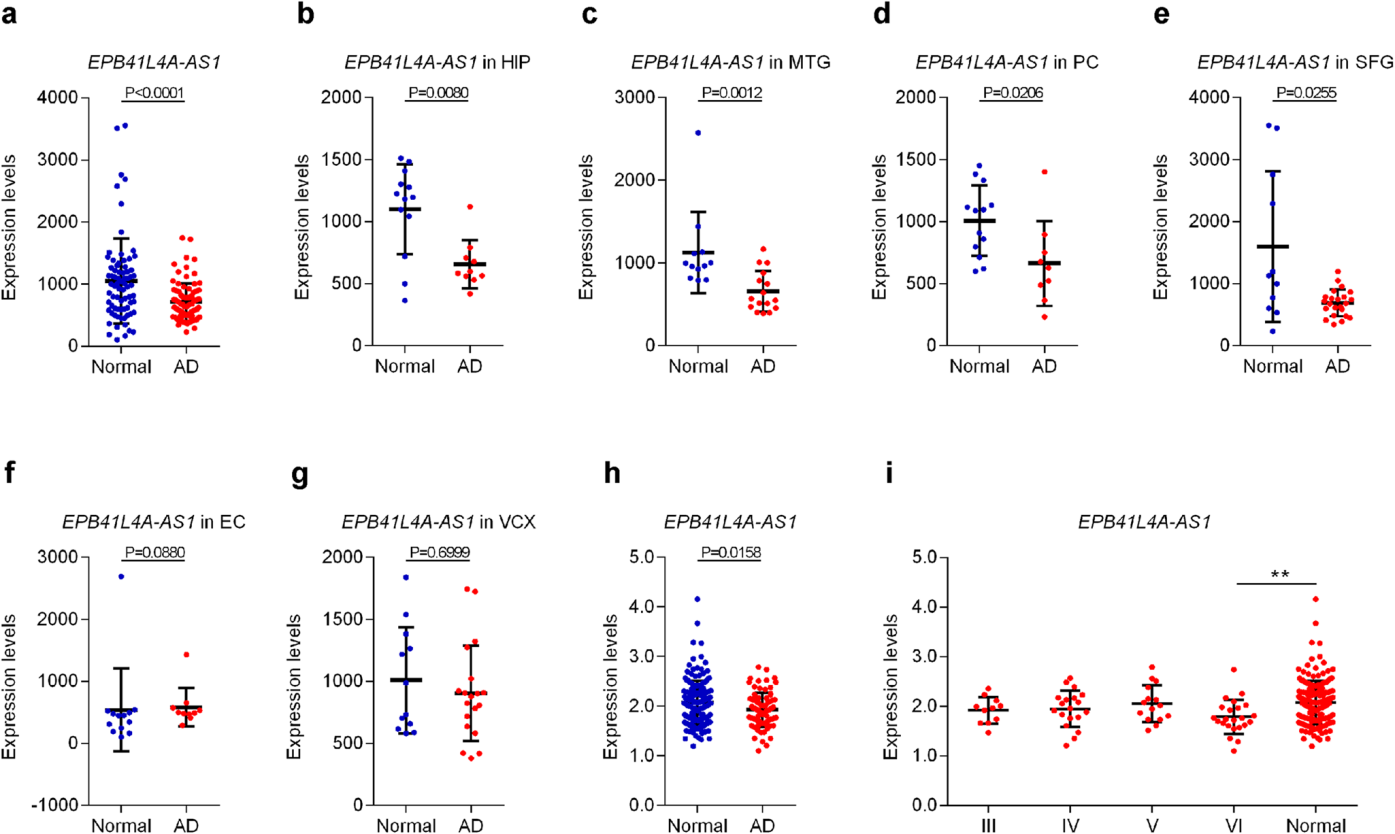
EPB41L4A-AS1 is an AD-related lncRNA. **a**–**g** EPB41L4A-AS1 expression in the different brain regions of patients with AD and normal controls in the AD dataset (GSE5281). *P*-values were calculated using the Mann-Whitney U test. **h**. EPB41L4A-AS1 expression in patients with AD and normal controls in the AD dataset (GSE48350). *P*-values were calculated using the Mann-Whitney U test. **i** EPB41L4A-AS1 expression in the hippocampi of patients with AD at different Braak stages and normal controls in the AD dataset (GSE48350). *P*-values were calculated using standard one-way ANOVA with Dunnett’s multiple comparisons test. **p < 0.01.

**Fig. 2:**
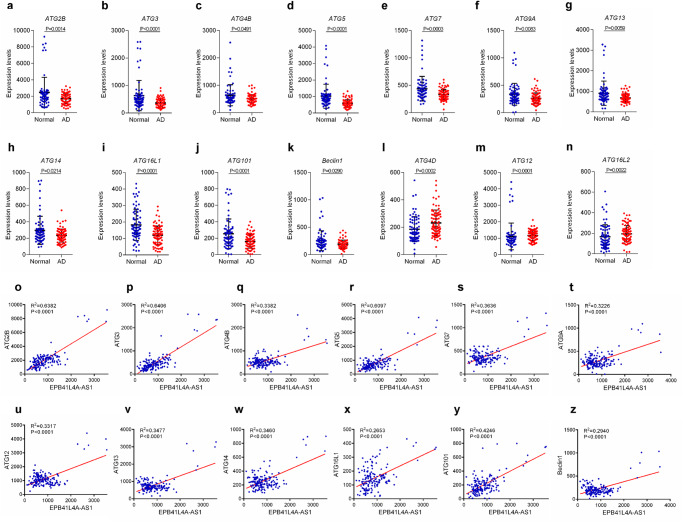
EPB41L4A-AS1 is associated with autophagy-related gene expression. **a**–**n** Differentially expressed autophagy-related genes in patients with AD and normal controls in the AD dataset (GSE5281). *P*-values were calculated using the Mann-Whitney U test. **o**-**z**. The correlation of EPB41L4A-AS1 and the differentially expressed autophagy-related gene expression in the AD dataset (GSE5281) was analyzed using linear regression.

We analyzed EPB41L4A-AS1 expression levels in different brain regions of patients with AD and normal controls using an AD dataset (GSE5281), and found that patients with AD showed significantly lower EPB41L4A-AS1 expression than that in normal controls (Fig. 1a), especially in the hippocampus (HIP), medial temporal gyrus (MTG), posterior cingulate (PC), and superior frontal gyru (SFG) (Fig. 1b–g). Moreover, we used another AD dataset (GSE48350) to analyze EPB41L4A-AS1 expression levels in AD hippocampi at different Braak stages. As shown in Fig. 1h and i, EPB41L4A-AS1 expression significantly decreased in AD hippocampi compared to that of normal hippocampi (Fig. 1h), especially in the hippocampal tissue at Braak stage VI (Fig. 1i). In addition, we found that multiple autophagy-related genes (ATGs), including ATG2B, ATG3, ATG4B, ATG5, ATG7, ATG9A, ATG13, ATG14, ATG16L1, ATG101, and Beclin1, had significantly lower expression in patients with AD (Fig. 2a–k), and ATG4D, ATG12, and ATG16L2 had significantly higher expression in patients with AD (Fig. 2l–n). Correlation analysis showed that EPB41L4A-AS1 was positively associated with the expression of ATG2B, ATG3, ATG4B, ATG5, ATG7, ATG9A, ATG12, ATG13, ATG14, ATG16L1, ATG101, and Beclin1 (Fig. 2o–z). Collectively, these results indicated that EPB41L4A-AS1 is an AD-related lncRNA associated with the expression of multiple autophagy-related genes.

### EPB41L4A-AS1 modulates Aβ clearance

**Fig. 3:**
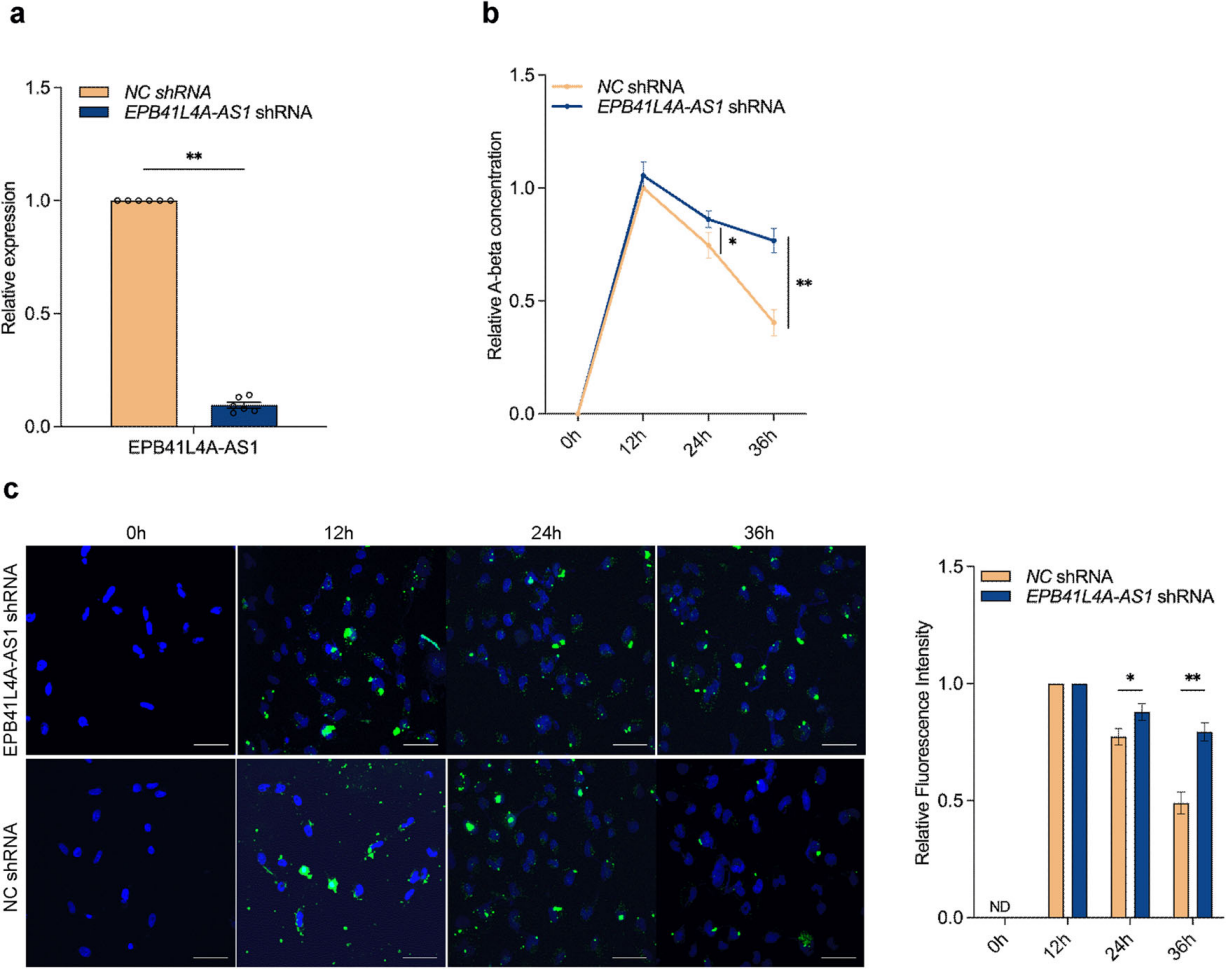
EPB41L4A-AS1 modulates Aβ clearance. **a** The relative expression of EPB41L4A-AS1 was analyzed through qPCR in U251 cells stably expressing EPB41L4A-AS1-target shRNAs (*EPB41L4A-AS1* shRNA cells) or negative control shRNAs (*NC* shRNA cells) in six independent experiments. *P*-values were calculated using the Mann-Whitney U test. **b** After Aβ_1-42_ was added to *EPB41L4A-AS1* shRNA U251 and *NC* shRNA U251 cells for the indicated time points, the relative concentrations of Aβ_1-42_ were analyzed through ELISA in three independent experiments. P-values were calculated using the multiple *t-*test. **c** After the addition of HiLyte Fluor™ 488-labelled Aβ_1-42_ for 0, 12, 24, and 36 h, *EPB41L4A-AS1* shRNA U251 and *NC* shRNA U251 cells were visualized through confocal microscopy. DAPI (blue) was used to stain the nuclei. Scale bar, 100 μm. the nuclei. The right histogram shows the relative fluorescence intensity of Aβ_1-42_. The fluorescence signal was determined using ImageJ software and normalized to a single cell. The relative fluorescence intensity of Aβ_1-42_ to that in 12 h was analyzed. *P*-values were calculated using the multiple *t*-test. The results are presented as the mean ± SD. ND: not detected. *p < 0.05, **p < 0.01, ***p < 0.001.

Because autophagy is an important pathway for Aβ clearance in neuroglial cells^7^, we further investigated whether EPB41L4A-AS1 downregulation in patients with AD was involved in Aβ clearance. We used lentivirus-based short hairpin RNA (shRNA) vectors to generate EPB41L4A-AS1-deficient human astrocytic U251 cells (*EPB41L4A-AS1* shRNA) and negative control cells (*NC* shRNA) (Fig. 3a). To evaluate the role of EPB41L4A-AS1 in Aβ clearance, we added Aβ_1-42_ to *EPB41L4A-AS1* shRNA and *NC* shRNA cell lines, harvested the cells at 0, 12, 24, and 36 h after Aβ_1-42_ incubation, and performed an enzyme-linked immunosorbent assay (ELISA). The Aβ_1-42_ concentration relative to that in *NC* shRNA cells at 12 h was calculated. The results showed that EPB41L4A-AS1 knockdown inhibited Aβ clearance (Fig. 3b). In addition, an immunofluorescence assay with anti-Aβ_1-42_ antibody was performed and similar results were obtained (Fig. 3c). Collectively, these results indicated that EPB41L4A-AS1 plays a vital role in neuroglial cell-mediated Aβ clearance.

### EPB41L4A-AS1 regulates multiple autophagy-related genes expression

**Fig. 4:**
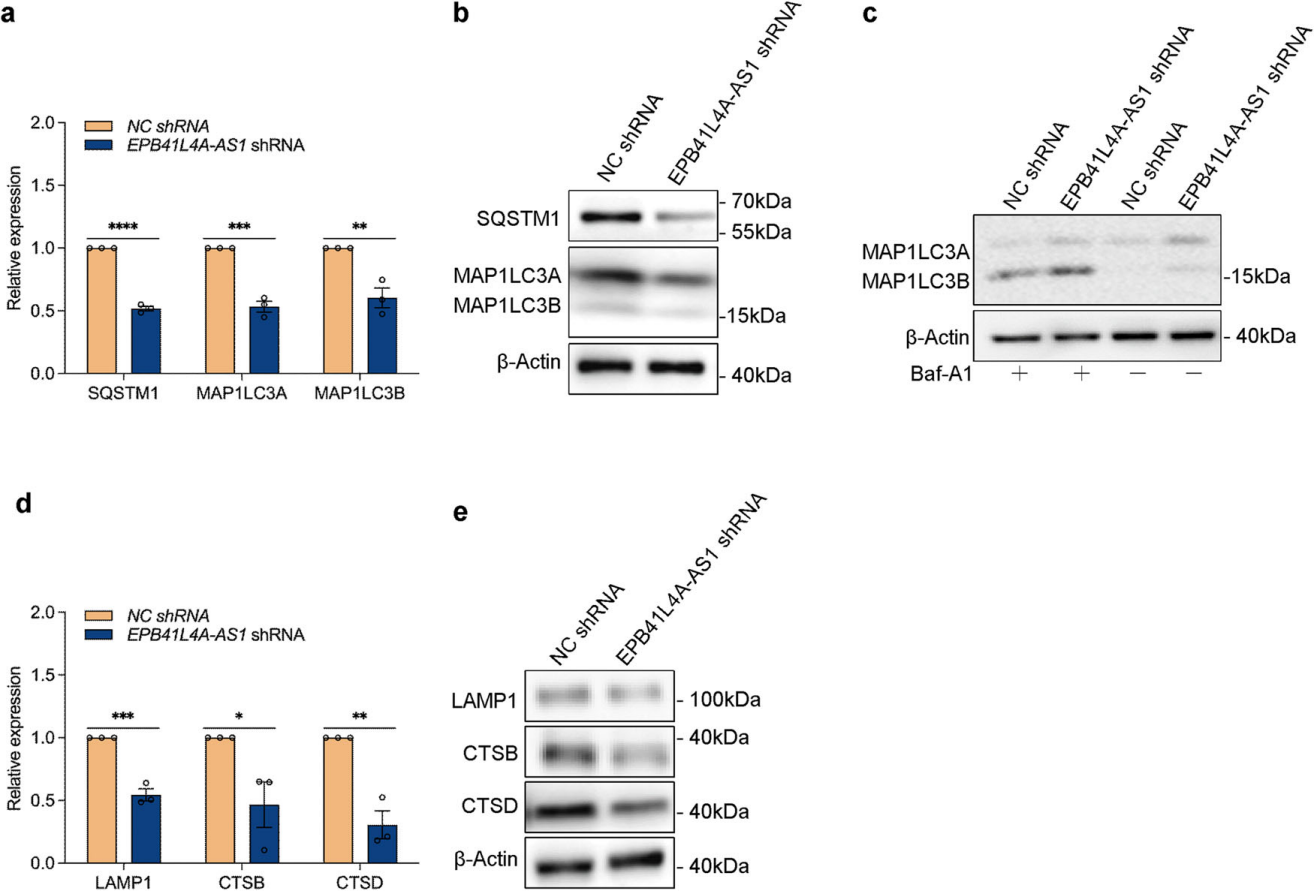
EPB41L4A-AS1 induces autophagy-lysosomal pathway. **a** The relative mRNA levels of the indicated autophagy marker genes in *EPB41L4A-AS1* shRNA U251 and *NC* shRNA U251 cells were analyzed using qRT-PCR in three independent experiments. *P*-values were calculated using the multiple *t*-test. **b**. The protein levels of the indicated autophagy marker genes and β-actin in *EPB41L4A-AS1* shRNA U251 and *NC* shRNA U251 cells were determined using western blotting. **c** After the addition of 100 nM Baf-A1 to *EPB41L4A-AS1* shRNA U251 and *NC* shRNA U251 cells for 24 h, The protein levels of MAP1LC3A, MAP1LC3B, and β-actin in cells were determined using western blotting. **d** The relative mRNA levels of the indicated lysosomal marker genes in *EPB41L4A-AS1* shRNA U251 and *NC* shRNA U251 cells were analyzed using qRT-PCR in three independent experiments. *P*-values were calculated using the multiple *t*-test. **e** The protein levels of the indicated lysosomal marker genes and β-actin in *EPB41L4A-AS1* shRNA U251 and *NC* shRNA U251 cells were determined using western blotting. The results are represented as the mean ± SD. *p < 0.05, **p < 0.01, ***p < 0.001, ****p < 0.0001.

As EPB41L4A-AS1 is associated with the expression of multiple autophagy-related genes (Fig. 2o–z), we next determined whether EPB41L4A-AS1 altered the autophagy markers SQSTM1 and MAP1LC3. The examination of the expression levels of SQSTM1, MAP1LC3A and MAP1LC3B in *EPB41L4A-AS1* shRNA and *NC* shRNA cells by quantitative real-time PCR (qRT-PCR) (Fig. 4a) and western blotting (Fig. 4b) showed that EPB41L4A-AS1 knockdown inhibited the expression of SQSMT1, MAP1LC3A, and MAP1LC3B in mRNA and protein levels. In addition, we added

Bafilomycin A1 (Baf-A1), an inhibitor of autophagy by disrupting autophagosome-lysosome fusion, to *EPB41L4A-AS1* shRNA and *NC* shRNA cells and found that EPB41L4A-AS1 knockdown decreased the protein levels of MAP1LC3A and MAP1LC3B with or without Baf-A1 (Fig. 4c), indicating that EPB41L4A-AS1 is required to maintain basal autophagy.

Because lysosomal degradation undergo autophagy-mediated Aβ clearance^16,17^, we further investigated effects of EPB41L4A-AS1 on expression levels of lysosomal markers, including lysosomal associated membrane protein 1 (LAMP1), Cathepsin B (CTSB), and Cathepsin D (CTSD). The results of qRT-PCR (Fig. 4d) and western blotting (Fig. 4e) showed that EPB41L4A-AS1 knockdown inhibited the expression of LAMP1, CTSB, and CTSD. These findings suggested EPB41L4A-AS1 plays a protective role in clearing Aβ by promoting autophagy-lysosomal pathway.

**Fig. 5:**
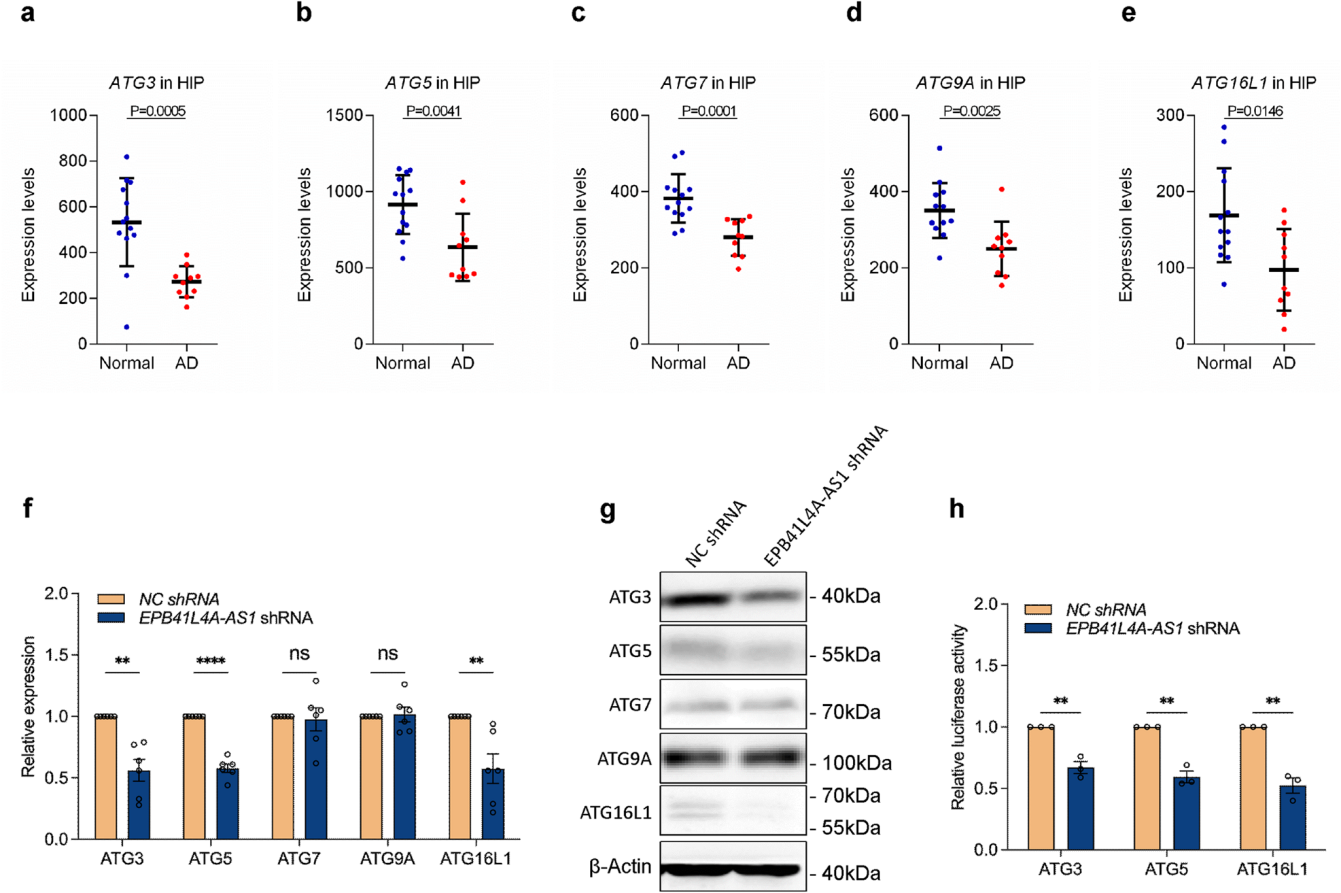
EPB41L4A-AS1 modulates autophagy-related gene transcription. **a**–**e** Differentially expressed autophagy-related genes in the hippocampus of patients with AD and normal controls in the AD dataset (GSE5281). *P*-values were calculated using the Mann-Whitney U test. **f** The relative mRNA levels of the indicated autophagy-related genes in *EPB41L4A-AS1* shRNA U251 and *NC* shRNA U251 cells were analyzed using qRT-PCR in six independent experiments. *P*-values were calculated using the multiple *t*-test. **g** The protein levels of the indicated autophagy-related genes and β-actin in *EPB41L4A-AS1* shRNA U251 and *NC* shRNA U251 cells were determined using western blotting. **h** After the transfection of the pGL3 enhancer plasmid containing the indicated autophagy-related gene promoters to *EPB41L4A-AS1* shRNA U251 and *NC* shRNA U251 cells for 36 h, the relative transcriptional activities of these promoters were determined with a luciferase assay in three independent experiments. *P*-values were calculated using the multiple *t*-test. The results are presented as the mean ± SD. ns: not significant. *p < 0.05, **p < 0.01, ***p < 0.001.

As hippocampus is closely associated with learning and memory and is affected in AD^18^, we next analyzed the abnormal expressed autophagy-related genes in hippocampus using the AD dataset (GSE5281) and found that the expression of ATG3, ATG5, ATG7, ATG9A, and ATG16L1 were downregulated in hippocampus in patients with AD (Fig. 5a–e). To confirm the association between EPB41L4A-AS1 and these autophagy-related genes, we performed qRT-PCR and western blotting to examine the expression of multiple autophagy-related genes in *EPB41L4A-AS1* shRNA and *NC* shRNA cells. The results showed that EPB41L4A-AS1 knockdown decreased the expression levels of ATG3, ATG5, and ATG16L1, but had no significant effect on ATG7 and ATG9A expression (Fig. 5f and g). It has been recently demonstrated that EPB41L4A-AS1 functions as a regulator of gene transcription^13–15^. To understand the mechanism by which EPB41L4A-AS1 regulates the expression of ATG3, ATG5, and ATG16L1 genes, luciferase assays were performed to determine whether EPB41L4A-AS1 directly regulates the transcriptional activities of these genes. Three luciferase reporters containing ATG3, ATG5, and ATG16L1 gene promoter fragments were used. The results showed that EPB41L4A-AS1 knockdown decreased the transcriptional activity of ATG3, ATG5, and ATG16L1 promoters (Fig. 5h). These results suggest that EPB41L4A-AS1 is required to maintain basal autophagy by regulating multiple autophagy-related genes expression

### EPB41L4A-AS1 regulates histone modifications located at the promoter region of autophagy-related genes

**Fig. 6:**
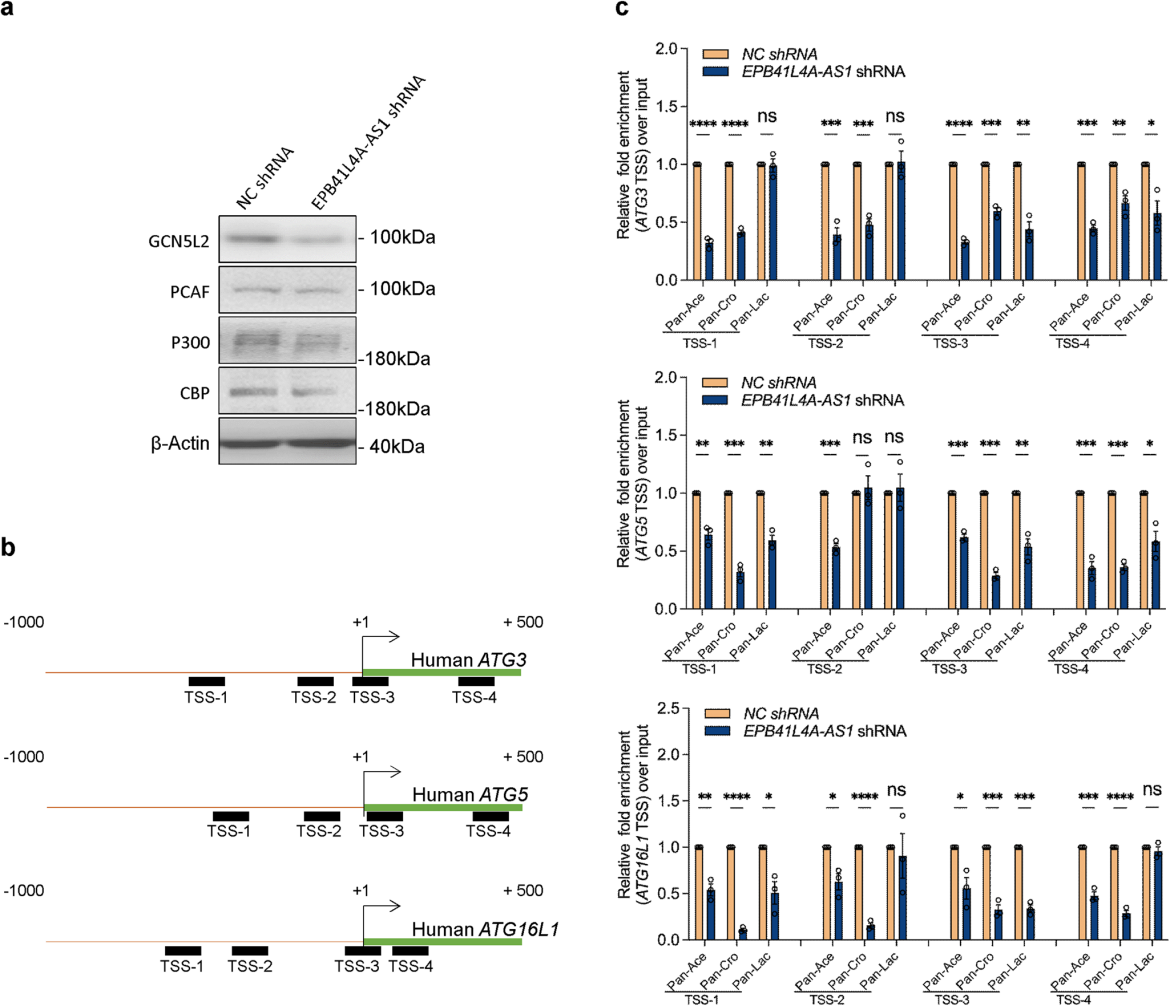
EPB41L4A-AS1 regulates multiple histone modifications in the TSSs region of target genes. **a** The protein levels of GCN5L2, P300, CBP, PCAF, and β-actin in *EPB41L4A-AS1* shRNA U251 and *NC* shRNA U251 cells were determined using western blotting. **b** A Schematic diagram shows the gene structure of ATG3, ATG5, and ATG16L1 genes, in which the black boxes represent the primer-amplified regions. **c** *EPB41L4A-AS1* shRNA U251 and *NC* shRNA U251 cells were collected for ChIP assays to analyze the relative fold-enrichment of ATG3, ATG5, and ATG16L1 promoters using anti-acetyl-lysine (Pan-Ace), crotonyl-lysine (Pan-Cro), or-L-lactate lysine (Pan-Lac) antibodies. Data points represent mean values determined from three independent experiments. *P*-values were calculated using the multiple *t*-test. The results are presented as the mean ± SD. ns not significant. *p < 0.05, **p < 0.01, ***p < 0.001.

Recently, EPB41L4A-AS1 was shown to interact with GCN5L2, an important histone acetyltransferase, to regulate histone crotonylation^14^. In this study, we performed western blotting to determine whether EPB41L4A-AS1 directly regulated the expression levels of GCN5L2 and other histone acetyltransferases. As shown in Fig. 6a, EPB41L4A-AS1 silencing decreased the expression levels of GCN5L2 but had no significant effect on P300, CBP, and PCAF. As GCN5L2 has been reported to participate in histone acetylation, crotonylation, and lactylation^14,19,20^ we performed chromatin immunoprecipitation (ChIP) experiments using antibodies against acetyl-lysine (Pan-Ace), crotonyl-lysine (Pan-Cro), and L-lactate lysine (Pan-Lac). To identify these modified histone-binding sites within the ATG3, ATG5, and ATG16L1 gene promoter sequences, we designed sets of primer pairs (Table S1) that recognized the TSSs regions of these genes (Fig. 6b). The ChIP assay demonstrated that knocking down EPB41L4A-AS1 decreased the enrichment of histone acetylation, crotonylation, and lactylation at the ATG3, ATG5, and ATG16L1 gene promoters (Fig. 6c). These results suggested that EPB41L4A-AS1 regulates histone modifications during gene regulation.

### Histone crotonylation and lactylation promote autophagy-related gene expression

**Fig. 7:**
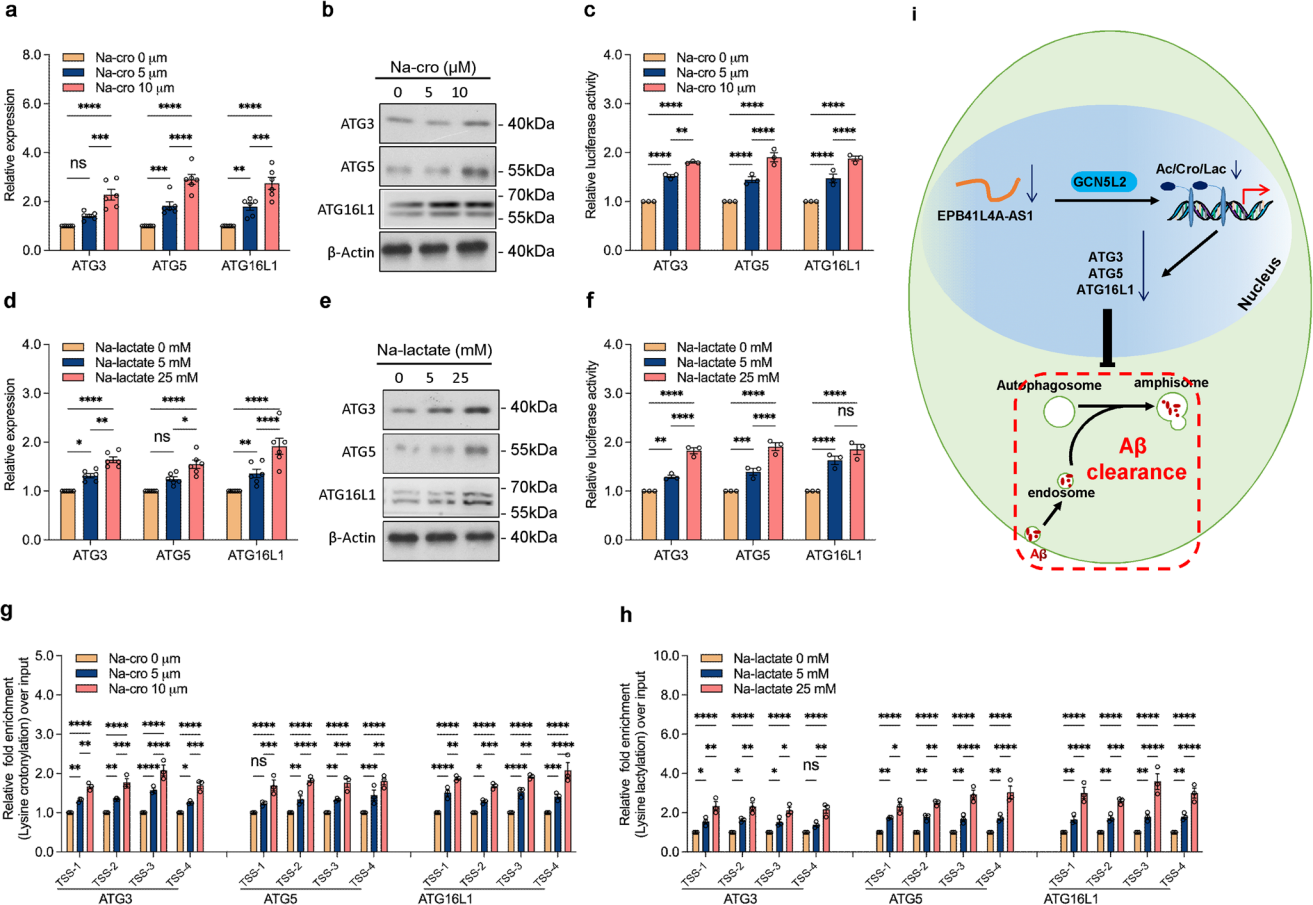
Histone crotonylation and lactylation promote autophagy-related gene expression. After incubation with indicated concentrations of Na-cro (**a**) or Na-lactate (**d**) for 24 h, the relative mRNA levels of indicated autophagy-related genes in U251 cells were analyzed using qRT-PCR in six independent experiments. P-values were calculated using the two-way ANOVA and Tukey’s multiple comparisons test. After incubation with indicated concentrations of Na-cro (**b**) or Na-lactate (**e**) for 24 h, the protein levels of indicated autophagy-related genes and β-actin in U251 cells were measured through western blotting. After the transfection of the pGL3 enhancer plasmid containing the indicated autophagy-related gene promoter to U251 cells for 12 h, the cells were incubated with indicated concentrations of Na-cro (**c**) or Na-lactate (**f**) for 24 h. The relative transcriptional activities of these promoters were determined with a luciferase assay in three independent experiments. P-values were calculated using the two-way ANOVA and Tukey’s multiple comparisons test. After incubation with the indicated concentrations of Na-cro (**g**) or Na-lactate (**h**) for 24 h, U251 cells were collected for ChIP assays to analyze the relative fold enrichment of the indicated autophagy gene promoters using crotonyllysine (**g**) and L-lactyl lysine (**h**) antibodies, respectively. The data points represent mean values determined from three independent experiments. *P*-values were calculated using the two-way ANOVA and Tukey’s multiple comparisons test. **i** Schematic model of the role of EPB41L4A-AS1 in Aβ clearance. The results are presented as the mean ± SD. ns not significant. *p < 0.05, **p < 0.01, ***p < 0.001.

As histone crotonylation and lactylation are two newly identified modifications with unknown functions^21,22^, we investigated their effects on autophagy-related gene expression. We added different concentrations of Na-crotonate (Na-cro) and Na-lactate to the culture media to assess the effects of changes in histone crotonylation and lactylation on target genes. The results of the qRT-PCR and western blotting revealed that the addition of exogenous Na-cro (Fig. 7a and b) and Na-lactate (Fig. 7d and e) significantly promoted the expression of ATG3, ATG5, and ATG16L1. The luciferase assay showed that the addition of exogenous Na-cro (Fig. 7c) and Na-lactate (Fig. 7f) increased the transcriptional activity of these gene promoters; and the results of the ChIP assay showed that the addition of exogenous Na-cro (Fig. 7g) and Na-lactate (Fig. 7h) increased lysine crotonylation and lactylation of these genes promoters, respectively. These results showed that histone crotonylation and lactylation stimulate autophagy-related gene transcription, indicating that histone crotonylation and lactylation may serve as markers for actively transcribed genes.

## Discussion

AD is a neurodegenerative disease with the histopathological hallmarks of the deposition of Aβ-containing neuritic plaques, hyperphosphorylation of Tau protein, and loss of neurons; the deposition of Aβ is considered the molecular driver of AD progression^4,5^. Although numbers of Aβ-based therapies have attempted to treat patients with AD, most of the clinical trials have shown that the effect of these therapies is mainly to improve cognitive and memory impairment and cannot prevent or delay the progression of the disease^23,24^. This may be because the pathogenesis of AD is complex and it is difficult for drugs targeting the Aβ peptide or its aggregates to effectively control or cure AD^25^. Thus, understanding the underlying mechanisms of the imbalance of Aβ generation and clearance and identifying novel molecular targets warrant further research.

lncRNAs are a class of functional ncRNAs containing more than 200 nucleotides that play significant roles in gene regulation by interacting with transcriptional factors, epigenetic alteration, and sequestration of RNA or protein^26^^.^ Growing evidence suggests that lncRNAs participate in various physiological and pathological processes^27–34^, including AD^35–37^. Since the identification of EPB41L4A-AS1 as lncRNA, various biological functions have been reported, including regulation of glucose metabolism leading to cancers^13^, T2DM^14^, early recurrent miscarriage^38^, and participating in the expression of a variety of cancer-related genes as competing endogenous RNAs^8,39–41^.

Defective autophagy has recently emerged as a causative factor in neurodegenerative disorders^42^. Autophagy plays a role in removing intracellular Aβ and MAPT/TAU tangles^7,43,44^ in patients with AD. In this study, we found that multiple autophagy-related genes exhibited lower expression levels in patients with AD compared to those in normal controls and that EPB41L4A-AS1 was associated with the downregulation of these autophagy-related genes. Therefore, we investigated the role of EPB41L4A-AS1 in the pathogenesis of AD. In this study, we found that lncRNA EPB41L4A-AS1 expression is downregulated in patients with AD, which inhibited the transcription of multiple autophagy-related genes by decreasing the expression level of histone acetyltransferase GCN5L2 and thus inhibiting the enrichment of histone acetylation, crotonylation, and lactylation near the TSSs of these genes, resulting in an inhibition of the autophagy-mediated Aβ clearance (Fig. 7i).

As the most abundant glial cells in the central nervous system, astrocytes have been reported to play an essential role in Aβ clearance by endocytosis^45,46^ and producing enzymes for amyloid degrading^47^. In this study, we found that silencing EPB41L4A-AS1 expression inhibited astrocytes mediated-Aβ clearance by modulating autophagy. Autophagosome formation is a highly complex process and is executed by the sequential function of ATG proteins, in which multiple ATG proteins coordinate to participate in every step of autophagosome formation, including initiation, nucleation, membrane expansion, closure, and fusion^48,49^. In the current study, we found that EPB41L4A-AS1 modulates autophagy by regulating the expression of ATG3, ATG5, and ATG16L1, three ATG proteins that are responsible for LC3 lipidation^49^. To determine the regulatory mechanism of EPB41L4A-AS1 in autophagy-related gene expression, we investigated the effects of EPB41L4A-AS1 on the transcription of these genes and the status of histone modification, an important epigenetic process to control gene transcription^50–53^, near the TSSs of these genes and found that EPB41L4A-AS1 positively regulated the transcriptional activities of these autophagy-related genes by altering the enrichment of histone acetylation, crotonylation, and lactylation located at their promoters. As histone crotonylation and lactylation are two newly identified histone modification types^21,22^, we determined their effects on target gene expression and found that histone crotonylation and lactylation correlated with the transcriptional activation of these autophagy-related genes. EPB41L4A-AS1 has been reported to alter histone crotonylation by interacting with the histone acetyltransferase GCN5L2^14^, a writer of histone crotonylation and lactylation^14,19^. Therefore, we investigated the effects of EPB41L4A-AS1 on the expression of GCN5L2 and other histone acetyltransferases, such as P300, CBP, and PCAF. We found that EPB41L4A-AS1 positively regulated GCN5L2 expression. These results indicate that EPB41L4A-AS1 plays a protective role in the autophagic degradation of Aβ.

As lysosomal degradation undergoes autophagy-mediated Aβ clearance^16,17^ and Aβ accumulation in AD brain may result from the dysfunction of the lysosomal pathway^54,55^, we thus confirmed whether EPB41L4A-AS1 downregulation influences lysosomal functions. The results showed that EPB41L4A-AS1 knockdown inhibited the expression of LAMP1, CTSB, and CTSD, the lysosomal markers, indicating that EPB41L4A-AS1 plays roles in maintaining lysosomal function. Taken together, these findings suggested that EPB41L4A-AS1 has a protective role in clearing Aβ by promoting the autophagy-lysosomal pathway. Increasing evidence demonstrated that the ubiquitin-proteasome pathway is another main intracellular degradation systems that play a vital role in protecting against the neurotoxicity of cytosolic Aβ accumulation^16,17^ and that the ubiquitin-proteasome pathway and autophagy-lysosomal pathway coordinate their actions in maintaining protein quality control by intersecting and communicating with each other^56^. Therefore, future investigation into the consequences and molecular mechanisms of EPB41L4A-AS1 downregulation in the ubiquitin-proteasome pathway should be conducted to understand the roles and underlying mechanisms of EPB41L4A-AS1 downregulation in Aβ accumulation. In addition, in the current study, we performed bioinformatics analysis and cell-based experiments to investigate the potential roles of EPB41L4A-AS1 in Aβ clearance. Therefore, further studies are needed to elucidate its roles using the AD mice model and potential roles in other AD pathogenesis, such as Tau hyperphosphorylation and aggregation.

In summary, this study reveals that EPB41L4A-AS1 is an AD-related lncRNA that mediates Aβ clearance by epigenetically regulating the expression of autophagy-related genes. These findings provide novel intervention targets for AD and other autophagy-related diseases.

## Methods

### Dataset

The Alzheimer’s Disease Datasets (GSE5281 and GSE48350) were downloaded from the Gene Expression Omnibus database (GEO, https://www.ncbi.nlm.nih.gov/gds/). EPB41L4A-AS1 and autophagy-related gene expression in AD patients and normal controls, and the correlation between EPB41L4A-AS1 and these autophagy-related genes expression was analyzed.

### Generation of the stable cell lines and cell culture

The lentivirus-based EPB41L4A-AS1-targeting shRNAs and negative control shRNAs were purchased from GenePharma Co. (Shanghai, China). Human astrocytic U251 cells were transiently transfected with these shRNAs, followed by puromycin selection to generate two stable monoclonal cell lines (*EPB41L4A-AS* shRNA cells and *NC* shRNA cells, respectively). These cells were grown in DMEM containing 10% foetal bovine serum and 10 U/ml of penicillin-streptomycin in a 5% CO_2_-humidified incubator at 37 °C.

### Aβ_1-42_ peptide quantitation by ELISA

To evaluate the roles of EPB41L4A-AS1 in Aβ clearance, *EPB41L4A-AS* shRNA cells and *NC* shRNA cells were inoculated with Aβ_1-42_ (MERCK, AG970-1MG). After 0, 12, 24, and 36 hours, the cells were washed and collected, and the Aβ_1-42_ levels were measured with the Amyloid Beta 42 Human ELISA Kit (Thermo Scientific, KHB3441) according to the manufacturer’s protocol. The data were normalized to the concentration of Aβ_1-42_ in the *NC* shRNA cells incubated with Aβ_1-42_ for 12 hours.

### Cell Transfection, RNA Isolation, Reverse Transcription and qPCR

For plasmid transfection, the cells were transiently transfected with Lipofectamine™ 3000 (Invitrogen, 1656200). Total RNA was isolated using RNAiso Plus (Takara, D9108B) and real-time qRT-PCR was performed using the ReverTra Ace® qPCR RT Master Mix with gDNA remover (TOYOBO, FSQ-301) and the SYBR Green PCR Master Mix (TOYOBO, QPK-201) according to the manufacturer’s protocol. All mRNA levels were normalized to β-Actin. The primers used are listed in Table S1.

### Western blotting

The cells were lysed in ice-cold whole cell extract buffer B (50 mM TRIS-HCl, pH 8.0, 4 M urea, and 1% Triton X-100) supplemented with protease inhibitor mixture. The cell extracts were resolved by SDS-PAGE and analyzed by western blotting. Protein bands were visualized using ECL Blotting Detection Reagents. All blots derive from the same experiment and they were processed in parallel. The antibodies used for western blotting include ATG3 (1:1000 dilution; 3415; Cell Signaling Technology), ATG5 (1:1000 dilution; 12994; Cell Signaling Technology), ATG7 (1:1000 dilution; 8558; Cell Signaling Technology), ATG9A (1:1000 dilution; 13509; Cell Signaling Technology), ATG16L1 (1:1000 dilution; 8089; Cell Signaling Technology), GCN5L2 (1:1000 dilution; 3305; Cell Signaling Technology), PCAF (1:1000 dilution; 3378; Cell Signaling Technology), P300 (1:1000 dilution; ab14984; Abcam), CBP (1:1000 dilution; ab253202; Abcam), SQSTM1 (1:1000 dilution; ab109012; Abcam), MAP1LC3A/B (1:1000 dilution; ab62721; Abcam), LAMP1 (1:1000 dilution; ab289548; Abcam), CTSB (1:1000 dilution; ab125067; Abcam), CTSD (1:1000 dilution; ab75811; Abcam), and β-Actin antibodies (1:1000 dilution; 60008-1-Ig; Proteintech).

### Immunofluorescence microscopy

To investigate the roles of EPB41L4A-AS in Aβ clearance, *EPB41L4A-AS* shRNA cells and *NC* shRNA cells were inoculated with Aβ_1-42_, HiLyte Fluor™ 488-labelled (ANASPEC, AS-60479-01). After 0, 12, 24, and 36 hours, the cells were washed, fixed, and counterstained with DAPI and observed under an Olympus FV1000 confocal laser microscope.

### Luciferase assay

For generation of luciferase reporters for the promoter assay, Three luciferase reporter constructs that contained inserted sequences from -500 bp to +500 bp relative to the TSSs of ATG3, ATG5, and ATG16L1 genes were purchased from Shanghai GenePharma Co., Ltd. The luciferase activity was measured using the Dual-Luciferase Reporter (DLR™) System (Promega, E1960) according to the manufacturer’s protocol with the ratio of Firefly to Renilla luciferase as an internal control for the transfection efficiency.

### ChIP assay

The ChIP assay was performed according to Dahl’s protocol^57^. Briefly, the cells were washed, fixed with 1% formaldehyde, and sonicated to shear the DNA. After centrifugation, the supernatants were incubated with antibodies against Acetyl-lysine (1:50 dilution; PTM-105RM; Jingjie PTM Biolab), Crotonyl-lysine (1:50 dilution; PTM-502; Jingjie PTM Biolab), and L-lactate lysine (1:50 dilution; PTM-1401RM; Jingjie PTM Biolab). Chromatin DNA was purified with protein G Dynabeads (Invitrogen, 10003D) and subjected to real-time qRT-PCR. The region-specific primers used are listed in Table S1.

### Statistics

The results are expressed as the mean ± SD from experiments repeated three or six times. Comparisons between the two groups were performed using a two-sample *t*-test. For three or more groups, multiple *t*-tests was conducted. A probability value < 0.05 was considered statistically significant. *p < 0.05, **p < 0.001, ***p < 0.0001, ****p < 0.00001.

### Reporting summary

Further information on research design is available in the Nature Research Reporting Summary linked to this article.

### Supplementary information

Supplemental Material

Reporting Summary

## Data Availability

The data that support the findings of this study are available from the corresponding author upon reasonable request.
