# Supplementary material for: EPB41L4A-AS1 is required to maintain basal autophagy to modulates Aβ clearance
Source: NPJ Aging. 2024 May 4;10(1):24. doi: 10.1038/s41514-024-00152-6 (PMC11069514; doi:10.1038/s41514-024-00152-6)
Supplement: Supplementary file 1 — Supplemental Material [file 41514_2024_152_MOESM1_ESM.pdf]

Supplementary Materials

Supplementary Figures

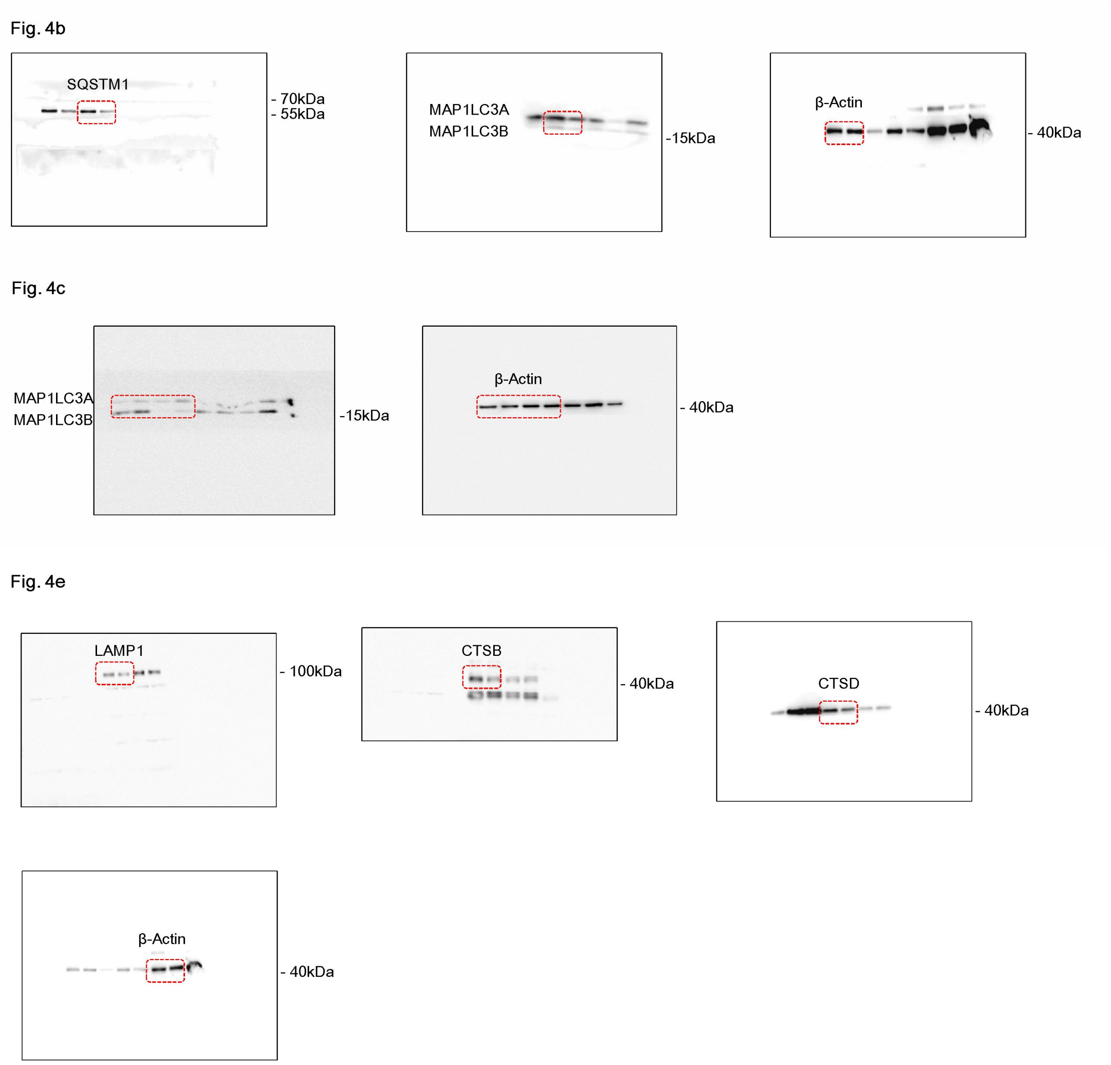

Supplementary Figure 1. Uncropped and unedited blot images related to Figure 4.

Fig. 5g

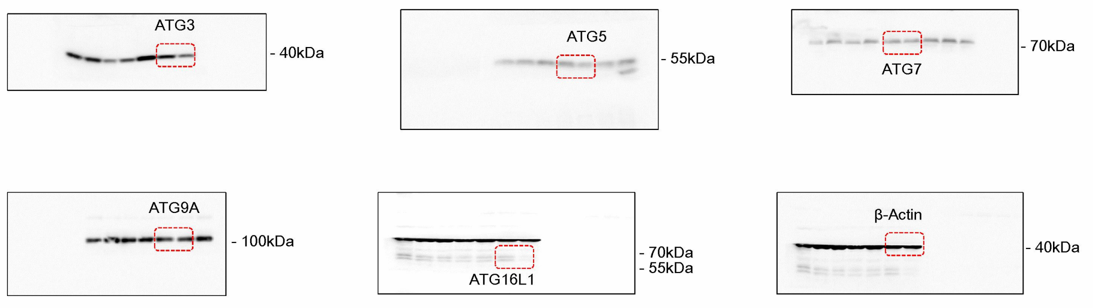

Supplementary Figure 2. Uncropped and unedited blot images related to Figure 5.

Fig. 6a

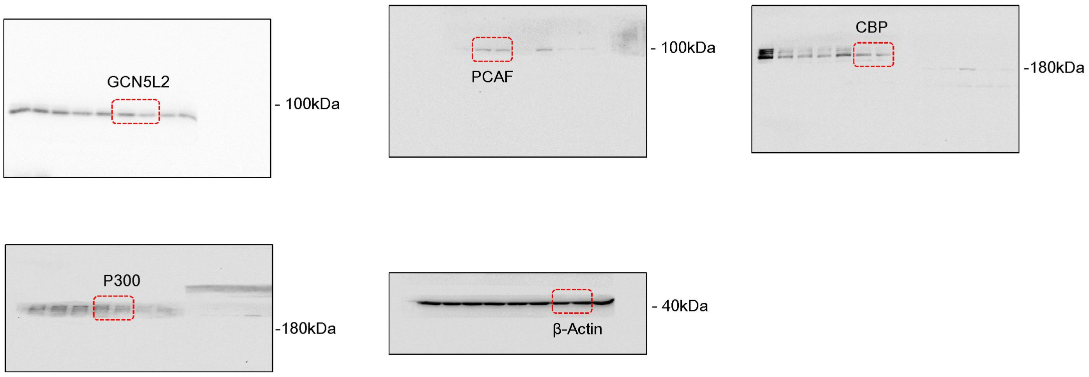

Supplementary Figure 3. Uncropped and unedited blot images related to Figure 6.

Fig. 7b

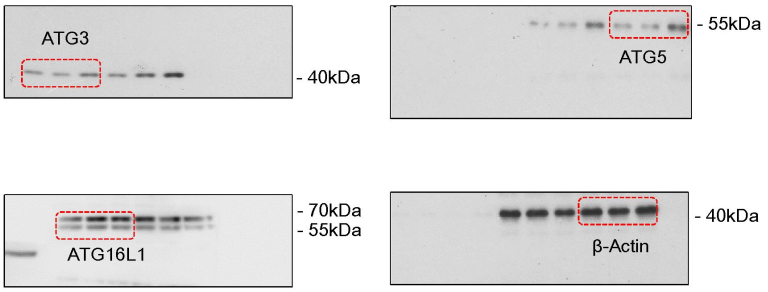

Fig. 7e

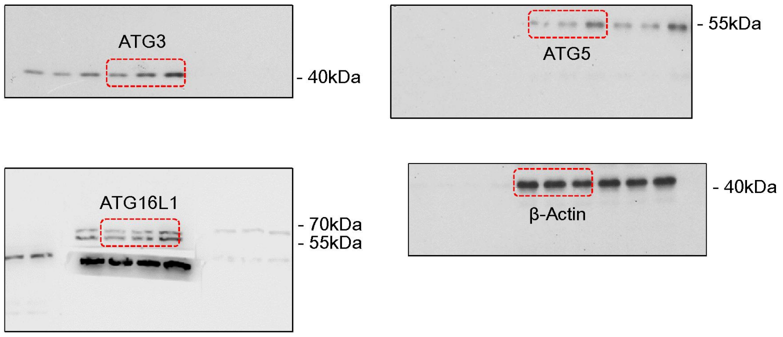

Supplementary Figure 4. Uncropped and unedited blot images related to Figure 7.

Supplementary tables

Supplementary Table 1. Sequences of primers used in this study.

Primer	Sense Sequence	Anti-sense Sequence
**Primers pairs for Real Time PCR**		
EPB41L4A-AS1	CCTGGTTTTATTTTCGTCA	ATCCATCTTCCACCTGTAG
ATG3	GATGGCGGATGGGTAGATACA	TCTTCACATAGTGCTGAGCAATC
ATG5	GTTTTGGGCCATCAATCGGAA	TCTCCTAGTGTGTGCAACTGT
ATG7	GGATGAAGCTCCCAAGGACAT	CCAGCAGAGTCACCATTGTAGTA
ATG9A	GCTCCCTGAAAATGCCCTCT	TTTGCGATAAGGCTCAGGGG
ATG16L1	ACACAAGAAACGTGGGGAGT	CTCCGTCTCCAGGTCAGAGA
MAP1LC3A	TCAGACCGGCCTTTCAAGCA	CGATGATCACCGGGATTTTGC
MAP1LC3B	CGAACAAAGAGTAGAAGATGTCCG	TGAGCTGTAAGCGCCTTCTA
SQSTM1	GGGGCTTGAGAAAGGATGAGG	GCCATCGCAGATCACATTGG
LAMP1	CACACCTTTTCCCCAATGCG	AAAGGTACGCCTGGATGGTG
CTSB	GCTTCGATGCACGGGAACAATG	CATTGGTGTGGATGCAGATCCG
CTSD	GACATCCACTATGGCTCGGG	TTGGCTGCGATGAAGGTGAT
β-Actin	TGACGTGGACATCCGCAAAG	CTGGAAGGTGGACAGCGAGG
**Primer pairs for ChIP**		
ATG3 TSS-1	GCGCATGTGTGACTAGAAGG	GCCAATACTGACAGCCCCATT
ATG3 TSS-2	CGGAAGCGTAACACTGAACG	GGGTCCATACGTGGAGAAGC
ATG3 TSS-3	TCCTCGCTTTGCTTCACTCG	CACGTGCCGGGAGAGTCTA
ATG3 TSS-4	TCACATTCTGCATCCTGGGG	GCAGCGAGGACATTTTCTGAC
ATG5 TSS-1	ACTGCCTCCCTAGAGCTTGAT	GTACTCTTGGATGGGTGGGAG
ATG5 TSS-2	CCTCCGTGTTCTGCCTAACC	CCTCCCCAAGCCCCAATAAC
ATG5 TSS-3	GTAGCAGGACTCCAGGAAGC	GAGGGTGACTGGACTTGTGG
ATG5 TSS-4	GTCTCGCCAACTCCACCTTG	TGTGTCCACCTTGTGTGAGG
ATG16L1 TSS-1	TAGGCCTTGGGAAGACACCA	AGGAACAAATGGGGTTCCCT
ATG16L1 TSS-2	TGGAGGGAACCCCATTTGTT	CGTTTCCGCCTGACTGCTTA
ATG16L1 TSS-3	TGACGCTTCCGGCTACAGG	GCAGCATGAAGCAACCAGC
ATG16L1 TSS-4	AGCAAGTGACATGTCGTCGG	GGATGATCTCCTCGAACGCC
